# Free flap salvage via direct urokinase injection into thrombosed veins: a case report

**DOI:** 10.1080/23320885.2025.2545195

**Published:** 2025-08-14

**Authors:** Masanobu Hayashi, Koreyuki Kurosawa, Toshiro Imai, Hiromu Matsunaga, Shinyo Ishi, Yoshimichi Imai

**Affiliations:** a Department of Plastic and Reconstructive Surgery, Tohoku University Graduate School of Medicine, Sendai, Miyagi, Japan; b Department of Plastic and Reconstructive Surgery, Miyagi Cancer Center, Natori, Miyagi, Japan; c Department of Plastic and Reconstructive Surgery, Sendai Medical Center, Sendai, Miyagi, Japan

**Keywords:** Venous thrombosis, thrombolytic therapy, urokinase, free flap

## Abstract

Intraoperative use of urokinase is a recognized method for salvaging compromised free flaps. However, protocols for dosage and administration vary, and no consensus exists regarding the optimal technique. Herein, we report a case of postoperative venous thrombosis in a free fibular flap. Despite the unsuccessful intra-arterial administration of urokinase owing to an extensive venous thrombus, we attempted to dissolve the thrombus through direct intravenous infusion using a 27 G needle at multiple sites in the vein where the thrombus had formed. Ten minutes after direct injection into the venous thrombus, venous blood flowed out and successful thrombolysis was achieved. Re-anastomosis was performed, leading to full use of the skin flap without partial necrosis. No hemorrhagic complications were observed. Intra-arterial injection of urokinase is an effective method of thrombolytic therapy for flap salvage. However, when the vein is completely occluded by thrombus, intraflap circulation of the agent *via* arterial infusion becomes difficult. Direct injection of urokinase into the occluded vein may serve as a potential method for resolving venous obstruction within the limited ischemic time of the flap.

## 1. Introduction

**Figure 1.**
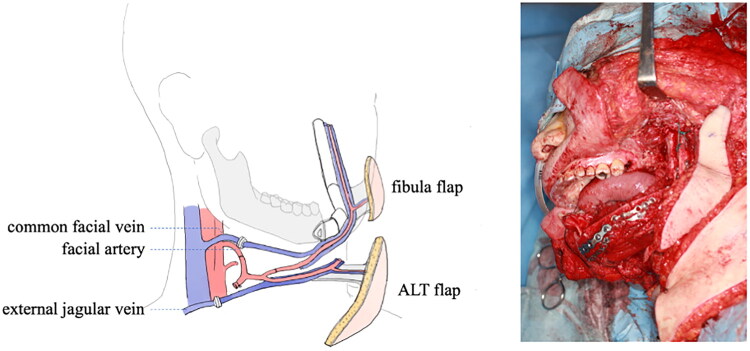
Post-reconstruction condition and schema of reconstruction.

Skin flap transplantation is a well-established microsurgical procedure that has advanced significantly over the years. Despite these advancements, complications, particularly flap necrosis attributed to anastomotic thrombus, have been reported in 1-5% of cases [1]. Thrombolytic therapy has been reported as an effective treatment for myocardial infarction and has also demonstrated efficacy in the management of other thrombotic conditions [2]. Its application has been extended to free flap salvage, with agents such as urokinase showing favorable outcomes; however, the optimal method and route of administration have yet to be established. This study presents a notable case of venous thrombus following free skin flap reconstruction (Figure 1) where salvage was achieved through the direct injection of urokinase into the site of venous thrombus formation. The subsequent sections outline the distinctive features of urokinase administration and provide a review of the pertinent literature.

## 2. Case presentation

A 41-year-old woman diagnosed with mandibular gingival carcinoma (cT4N2cM0) involving the left buccal soft tissue underwent surgery by an otolaryngologist that included mandibulectomy with removal of the mandibular head and bilateral cervical dissection. Reconstruction of the tissue defect was undertaken by a plastic surgeon using a combination of free fibula and anterolateral thigh (ALT) flaps. Preoperative laboratory investigations revealed no abnormalities in platelet count or coagulation parameters. The results were as follows: platelet count, 352 × 10³/μL; activated partial thromboplastin time (APTT), 31.4 s; and prothrombin time–international normalized ratio (PT–INR), 0.96.

The recipient vessel for the ALT flap was the facial artery, with an arterial anastomosis. The external femoral circumflex vein was sutured to the external jugular vein using an automated anastomotic device. For the fibular flap, the fibular artery was sutured to the artery of the vastus lateralis branch of the ALT flap. All arterial anastomoses were performed in an end-to-end fashion using 9-0 non-absorbable monofilament sutures. While two fibular veins were observed, only one venous anastomosis was performed in an end-to-end fashion to the right common facial vein using an automated anastomosis device (φ 2.5 mm). Circulation resumed successfully with a robust blood flow to the skin flap. The flap inhibition times were 1 h and 10 min for the ALT flap and 2 h for the fibula flap.

## 3. Postoperative course and reoperation

**Figure 2.**
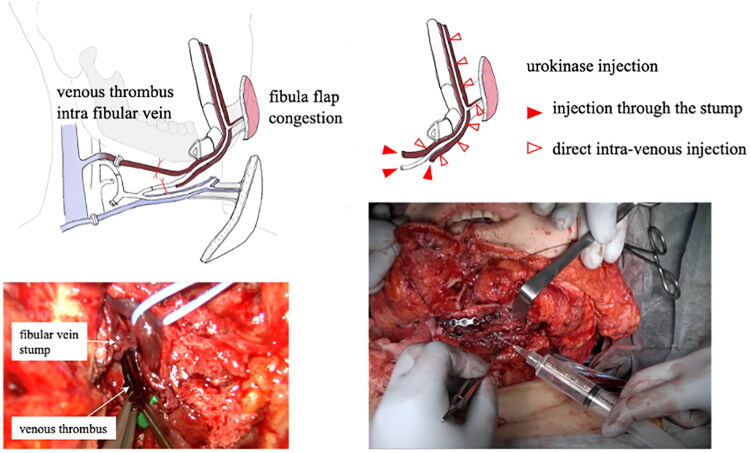
Red thrombus, extent of thrombus formation, and direct injection of urokinase into vein.

**Figure 3.**
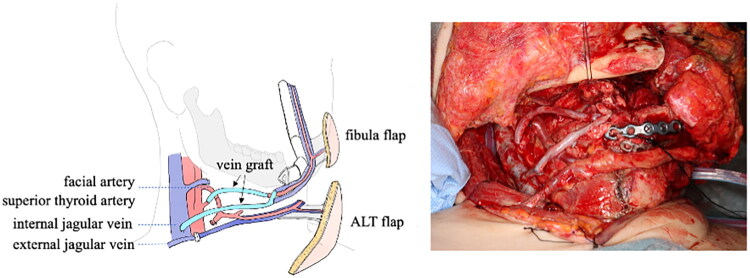
Post-revascularization condition and schema of reconstruction.

Twenty hours postoperatively, the patient developed multiple purpurae on the island of the fibula flap. Upon opening the neck in the ICU, thrombus formation (Figure 2) was observed in the fibular vein, filling the skin perforator branch of the skin island from the anastomosis (Figure 3). Considering the challenges involved in thrombus removal, we opted for thrombolytic therapy.

To avoid the systemic effects of thrombolytic therapy, both the venous and arterial anastomoses were disconnected prior to administration. Urokinase (60,000 IU) was dissolved in 100 mL of saline for injection. Initially, 30,000 IU of urokinase saline solution was administered intra-arterially through a branch of the fibular artery. With no venous blood outflow observed after 20 min, a decision was made to inject urokinase directly into the fibular vein at the site of thrombus formation. To minimize bleeding from the puncture site, using a fine 27 G needle, 30,000 IU urokinase was injected into the vein at multiple points from the peripheral to the central part of the thrombus formation site, 25 min after arterial infusion (Figure 2).

Ten minutes after intra-venous infusion, venous blood began to flow out of the veins, allowing heparin to circulate through the vessels within the skin flap. Reanastomosis was then carried out.

The recipient artery was secured by dissecting the previously unused right superior thyroid artery. The fibular artery was anastomosed to the right superior thyroid artery using the small saphenous vein as a vein graft. Concurrently, the fibular vein was anastomosed to the internal jugular vein using the small saphenous vein graft. Another fibular vein was also anastomosed to the internal jugular vein *via* a vein graft, with the two companion veins serving as drainage routes (Figure 3). All vascular reanastomoses, both arterial and venous, were performed in an end-to-end fashion using 9-0 non-absorbable monofilament sutures.

Arterial thrombosis was observed after arterial anastomosis, requiring reanastomosis. After reanastomosis, arterial blood flow was restored through the skin flap. Salvage of the skin flap was then determined to be successful.

## 4. Follow up and outcomes

**Figure 4.**
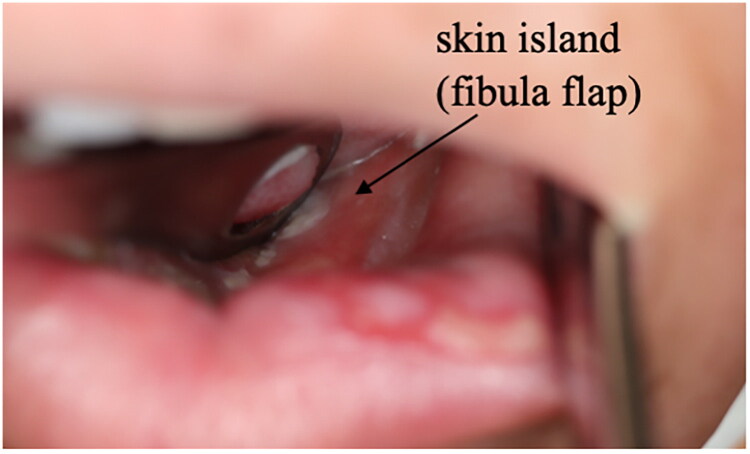
Fibula flap skin island 5 months after surgery (after irradiation).

Heparin administration at a dose of 10,000 IU/day was initiated for 14 days post-surgery. Subsequent follow-up assessments using ultrasound doppler showed no problem with blood flow to the skin flaps. Both the fibula and anterolateral thigh (ALT) flaps exhibited complete viability, including the island (Figure 4).

## 5. Discussion

Despite advancements in microsurgical techniques, reports indicate that 1-5% of skin flap necrosis results from an anastomotic thrombus [1]. The timely detection of impaired blood flow and prompt thrombus removal are imperative for successful flap salvage.

Thrombus formation is broadly categorized into arterial and venous thrombi, each exhibiting distinct characteristics. Arterial or white thrombi are formed through platelet aggregation and coagulation at the sites of intimal damage. In contrast, venous or red thrombi contain fibrin and red blood cells and are formed due to slower blood flow, reduced blood pressure, and bending [3,4].

In cases of anastomotic thrombus formation in free skin flaps, heparin administration or removal of the thrombus *via* forceps or a Fogarty catheter are common interventions. However, when microthrombi are present, complete removal becomes challenging. Thrombolytic therapy, such as the use of urokinase, is considered in such instances [5,6].

Thrombolytic therapy, initially employed in myocardial and cerebral infarctions, found its clinical application in free skin flap salvage by Lipton et al. in 1987 [7]. Subsequent studies reported the efficacy of plasminogen activators, including tissue-type plasminogen activator (t-PA), recombinant tissue plasminogen activator (rt-PA), and urokinase, for free skin flap salvage [8,9]. Plasminogen activators degrade plasminogen into plasmin, promoting a thrombolytic effect by breaking down fibrin into soluble fibrin degradation products. Notably, owing to their action on fibrin, plasminogen activators are considered more effective in dissolving venous thrombi that are primarily composed of fibrin clots [3].

Despite numerous clinical reports on urokinase efficacy for skin flap salvage, variations exist in the route, dosage, and administration method, as well as in the state of thrombus formation. The establishment of standardized indications, dosage, and administration methods remains elusive. Additionally, the risk of bleeding complications, a recognized side effect of thrombolytic therapy, warrants careful consideration. Table 1 presents a tabulation of studies on urokinase flap salvage involving intra-arterial infusion. In cases where the venous outflow tract is occluded during intra-arterial infusion, various approaches have been reported to ensure proper perfusion without leakage to peripheral arteries. These approaches include urokinase infusion through an opening in the arterial anastomosis [10–12], infusion through direct injection into the artery pedicle [5,13,14], infusion through an arterial branch [5], and the use of a pressurized bag [5]. The urokinase dosage varies widely, ranging from 60,000 to 400,000 IU, and the appropriate dosage has yet to be standardized. None of the studies reported systemic bleeding complications in the high-dose cases.

**Table 1.** •••.

Author, Year	Case	Salvage rate (%)	Indication for thrombolysis	Urokinase dosage (IU)	Injection techinique	Bleeding complication
Serletti, 1998	5	100	venous thrombosis	250000	arterial injection (direct injection to artery pedicle)	-
Darpa, 2005	2	100	venous thrombosis(1) intra-flap microthrombosis(1)	50000-100000	arterial injection (resected anastomosis)	-
Agostini, 2012	1	100	venous thrombosis	400000	arterial injection (direct injection to artery pedicle)	-
Shao-Yun, 2017	1	100	venous thrombosis	60000	artrial injection (resected anastomosis)	-
Namgoong, 2018	6	100	intra-flap thrombosis	100000	artrial injection (resected anastomosis)	-
Jun-Hyeok Kim, 2023	16	81.3	arterial thrombosis(1) venous thrombosis(10) arterial and venous thromobosis(5)	30000-100000	arterial injection(direct injection to artery pedicle)	-
Duncan, 2023	8	38	areterial thrombosis(4) venous thrombosis(3) intra-flap microthrombosis(1)	30000-200000	arterial injection (resected anastomosis, side branch)	-

The estimated half-life of urokinase is 16 min [15], suggesting that direct infusion into the thrombus formation site may have contributed to thrombolysis. In this case, despite a lower urokinase dose than in previous reports, the combination of intra-arterial and direct intra-venous infusion proved to be effective.

All previous reports on venous thrombolysis have utilized intra-arterial administration. However, the valvular structure of veins poses challenges for retrograde intra-venous infusion from the venous stump to dissolve the peripheral thrombus. In cases with interrupted venous outflow due to extensive thrombus formation, urokinase cannot reach the thrombus formation area, and this hinders the thrombolytic effect. Duncan et al. proposed a skin flap rescue protocol that entailed monitoring the effect of urokinase after 20 min, followed by additional urokinase injections based on the urokinase half-life, yet reported a skin flap rescue rate of 38% [6]. For effective dissolution of microthrombi within the flap, antegrade intravascular perfusion *via* arterial injection is essential. However, in the presence of venous thrombotic occlusion, intravascular perfusion through arterial injection is not feasible. Furthermore, given the critical time limitation imposed by flap ischemia, prompt resolution of venous obstruction is crucial for successful flap salvage. Although direct injection to the thrombus site, as in this case, has not been previously reported, it presents a direct approach for treating venous thrombi and may be considered in combination with a dynamic injection when needed. This method may be effective as one approach to resolving thrombotic occlusion within a limited time frame. However, it is also possible that the thrombolytic effect of the arterial injection manifested in a delayed manner. As this is a single case report, scientific evidence supporting the efficacy of this technique remains insufficient.

In general, systemic urokinase doses exceeding 500,000 IU per day may elevate the risk of bleeding [13]. However, in skin flap salvage, selective urokinase administration through the skin flap with vein disconnection minimizes the systemic effects. Previous reports have indicated no systemic bleeding complications even at doses of 1,000,000 IU [6]. In the present case, multiple vein stem punctures showed no adverse events, supporting the safety of urokinase administration.

For postoperative therapy, Serletti et al. have recommended prophylactic intra-venous urokinase use, whereas continuous intra-venous heparin has been commonly reported [13]. Postoperative systemic urokinase levels should be carefully monitored for bleeding risk. In the present case, systemic urokinase was not administered postoperatively.

## 6. Conclusion

The salvage of a skin flap *via* urokinase-mediated thrombolysis is a potent therapeutic modality, showcasing efficacy in thrombus dissolution. Its selective administration to the skin flap minimizes the risk of systemic bleeding complications. Direct injection of urokinase into the occluded vein may serve as a potential method for resolving venous obstruction within the limited ischemic time of the flap.
